# Identification of Potential Multitarget Compounds against Alzheimer’s Disease through Pharmacophore-Based Virtual Screening

**DOI:** 10.3390/ph16121645

**Published:** 2023-11-23

**Authors:** Géssica Oliveira Mendes, Moysés Fagundes de Araújo Neto, Deyse Brito Barbosa, Mayra Ramos do Bomfim, Lorena Silva Matos Andrade, Paulo Batista de Carvalho, Tiago Alves de Oliveira, Daniel Luciano Falkoski, Eduardo Habib Bechelane Maia, Marcelo Siqueira Valle, Laila Cristina Moreira Damázio, Alisson Marques da Silva, Alex Gutterres Taranto, Franco Henrique Andrade Leite

**Affiliations:** 1 Laboratory of Molecular Modeling, Department of Health, State University of Feira de Santana, Feira de Santana 44036-900, BA, Brazil; gomendes05@gmail.com (G.O.M.); moysesfagundes@gmail.com (M.F.d.A.N.); deyse.brito@hotmail.com (D.B.B.); mayramosbonfim@hotmail.com (M.R.d.B.); 2 Laboratory of Chemoinformatics and Biological Assessment, Department of Health, State University of Feira de Santana, Feira de Santana 44036-900, BA, Brazil; lorena.pharm@gmail.com; 3 Feik School of Pharmacy, University of the Incarnate Word, San Antonio, TX 78212, USA; pcarvalh@uiwtx.edu; 4 Department of Bioengineering, Federal University of São João del-Rei, São João del-Rei 36301-160, MG, Brazildlfalkoski@yahoo.com.br (D.L.F.); proftaranto@hotmail.com (A.G.T.); 5 Department of Informatics, Management and Design, Federal Center for Technological Education of Minas Gerais (CEFET-MG), Divinópolis 35503-822, MG, Brazil; habib@cefetmg.br (E.H.B.M.); alisson@cefetmg.br (A.M.d.S.); 6 Department of Natural Sciences, Federal University of São João del-Rei, São João del-Rei 36301-160, MG, Brazil; marcelovalle@gmail.com; 7 Department of Medicine, Federal University of São João del-Rei, São João del-Rei 36301-160, MG, Brazil; lailacmdamazio@gmail.com

**Keywords:** Alzheimer’s disease, human acetylcholinesterase, human butyrylcholinesterase, human beta-secretase 1

## Abstract

Alzheimer’s disease (AD) is a neurodegenerative disease characterized by progressive loss of cognitive functions, and it is the most prevalent type of dementia worldwide, accounting for 60 to 70% of cases. The pathogenesis of AD seems to involve three main factors: deficiency in cholinergic transmission, formation of extracellular deposits of β-amyloid peptide, and accumulation of deposits of a phosphorylated form of the TAU protein. The currently available drugs are prescribed for symptomatic treatment and present adverse effects such as hepatotoxicity, hypertension, and weight loss. There is urgency in finding new drugs capable of preventing the progress of the disease, controlling the symptoms, and increasing the survival of patients with AD. This study aims to present new multipurpose compounds capable of simultaneously inhibiting acetylcholinesterase (AChE), butyrylcholinesterase (BChE)—responsible for recycling acetylcholine in the synaptic cleft—and beta-secretase 1 (BACE-1)—responsible for the generation of amyloid-β plaques. AChE, BChE, and BACE-1 are currently considered the best targets for the treatment of patients with AD. Virtual hierarchical screening based on a pharmacophoric model for BACE-1 inhibitors and a dual pharmacophoric model for AChE and BChE inhibitors were used to filter 214,446 molecules by QFIT_BACE_ > 0 and QFIT_DUAL_ > 56.34. The molecules selected in this first round were subjected to molecular docking studies with the three targets and further evaluated for their physicochemical and toxicological properties. Three structures: ZINC45068352, ZINC03873986, and ZINC71787288 were selected as good fits for the pharmacophore models, with ZINC03873986 being ultimately prioritized for validation through activity testing and synthesis of derivatives for SAR studies.

## 1. Introduction

Alzheimer’s disease (AD) is a progressive and irreversible neurodegenerative disease that causes memory loss and several cognitive disorders [1]. AD is responsible for 70% of all cases of dementia and affects approximately 50 million people worldwide [2]. Those numbers are expected to double by 2050 [2].

### 1.1. Pathogenesis of Alzheimer’s Disease 

Alzheimer’s pathogenesis involves three main factors: the first is characterized by a deficiency in cholinergic transmission due to the selective loss of cholinergic neurons, the second is related to extracellular deposits of β-amyloid protein due to the catalytic action of beta-secretase 1 (BACE-1; EC 3. 4. 23. 46), and the third occurs through the formation of neurofibrillary clumps of a phosphorylated form of the TAU protein [1,3].

The cholinergic hypothesis is described as one of the main causes of AD [1]. Since the decline of cholinergic neurons induces a lack of acetylcholine (ACh), one way to compensate for this is to reduce the postsynaptic destruction of ACh by cholinesterases. Cholinesterases are a family of enzymes responsible for catalyzing the hydrolysis of ACh into choline and acetic acid to be reused into neuronal processes and are divided into two types: acetylcholinesterase (AChE; EC 3.1.1.7), and butyrylcholinesterase (BChE; EC 3.1.1.8). These enzymes are found mainly in the central nervous system, and their inhibition would lead to an increase in the available ACh at the synaptic cleft, promoting cognitive improvement [3].

The hypothesis of extracellular deposits of β-amyloid protein is noteworthy as it can explain the neurodegeneration process. This hypothesis states that the catalytic action of beta-secretase 1 generates, through the amyloidogenic pathway, the accumulation of insoluble β-amyloid peptide plaques in neurons. This pathophysiological process begins with the enzyme β-secretase (BACE1), which initiates the cleavage of the transmembrane protein called APP (Amyloid Precursor Protein). This cleavage is completed by another enzyme, γ secretase, generating the β-amyloid peptide (Aβ), which aggregates into oligomers, forming plaques that are deposited in different parts of the brain, mainly in the hippocampal neurons, basal nucleus, cortex entorhinal, and associative cortex [4].

### 1.2. Current Pharmacotherapy for Alzheimer’s Disease

The therapeutic resources currently available for AD include cholinesterase inhibitors (donepezil, rivastigmine, and galantamine), N-methyl-D-aspartate (NMDA) receptor antagonists (memantine), chelating agents (deferiprone), and metal–protein attenuating compounds (MPACs) (clioquinol) [1]. These drugs are essentially prescribed for symptomatic treatment and cannot prevent neurodegeneration. They also present serious adverse effects (e.g., hepatotoxicity, hypertension, and weight loss) and low therapeutic efficacy [4], giving urgency to the search for new drugs capable of preventing the disease’s progression and controlling its symptoms.

### 1.3. Multitarget Inhibitors

An approach that has been attracting interest for the treatment of multifactorial diseases such as AD is the identification and/or design of multitarget inhibitors, compounds capable of simultaneously acting on two or more biological targets, enhancing the therapeutic efficiency with lower doses. Previous studies show that treatment with multi-active drugs or molecular hybrids has greater efficacy and fewer adverse events [5] when compared with drug combination therapy in patients with complex diseases. This approach of using molecular hybrids has good potential to achieve the goal of slowing down the progression of AD [6].

One way to identify lead compounds with multitarget inhibition properties involves the use of computational strategies capable of prioritizing promising molecules for biological assays and accelerating the discovery of new, safe, and effective drugs. The ligand-based approach of computer-aided drug design can help to identify promising inhibitors with triple activity by defining the main stereo-electronic requirements of pharmacophore models for compounds with activity against each of the three molecular targets [7]. Ligand-based approaches have shown high efficiency in screening large datasets with low computational costs [8]. The selected compounds can be further subjected to structure-based approaches (e.g., molecular docking), and those showing complementarity with the target site are refined, helping to hypothesize the binding modes responsible for biological activity [9].

This study aimed to build and validate pharmacophore models of BACE-1, AChE, and BChE inhibitors and use them for a joint hierarchical virtual screening, followed by molecular docking virtual screening. Those models were used to identify promising multitarget inhibitors of beta-secretase 1, acetylcholinesterase, and butyrylcholinesterase in a Sigma-Aldrich^®^ (St. Louis, MO, USA) dataset.

## 2. Results and Discussion

Computational methods used to mine for potentially active compounds have presented a quicker enrichment rate of potential candidate molecules for biological testing than random methods. Their capacity for building pharmacophore models can quickly identify essential stereo-electronic requirements for inhibition of specific targets and help to prioritize potentially active compounds.

### 2.1. Pharmacophore Model Generation and Evaluation

The search parameters for potential BACE-1 inhibitors were based on the main pharmacophore features of known active inhibitors and were used to identify molecules sharing the same stereo-electronic features. In total, 56 inhibitors were chosen with an IC_50_ equal or lower than 1000 nM, with 14 of them being used to generate the model and the remaining 42 for the validation stage.

The heuristic of the Genetic Algorithm (GA) generated ten pharmacophore models, and their parameters are presented in Table 1. Those internal statistical parameters are expected to fit a strain energy criterion lower than 100.0 kcal/mol [10], as high energy values (>100.00 Kcal/mol) reflect a high conformational tension, building energetically unfavorable conformers [11]. Therefore, models 05, 06, 07, and 10 were discarded.

All models were evaluated for the PARETO value, which considers the listed parameters Mol_qry, H_bond, Sterics, and Energy. The conformers are overlapped and the pharmacophore agreement for the generation of the models is directly linked to the quality of pharmacophoric models [12]. All models, even the rejected ones, had PARETO = 0, meaning all the models were statistically similar. The parameters provided by GALAHAD were not sufficient to define the best model, and another metric, the Receiver Operation Characteristic (ROC) curves, was calculated to assess the ability of the models to differentiate between real and false positives.

**Figure 1:**
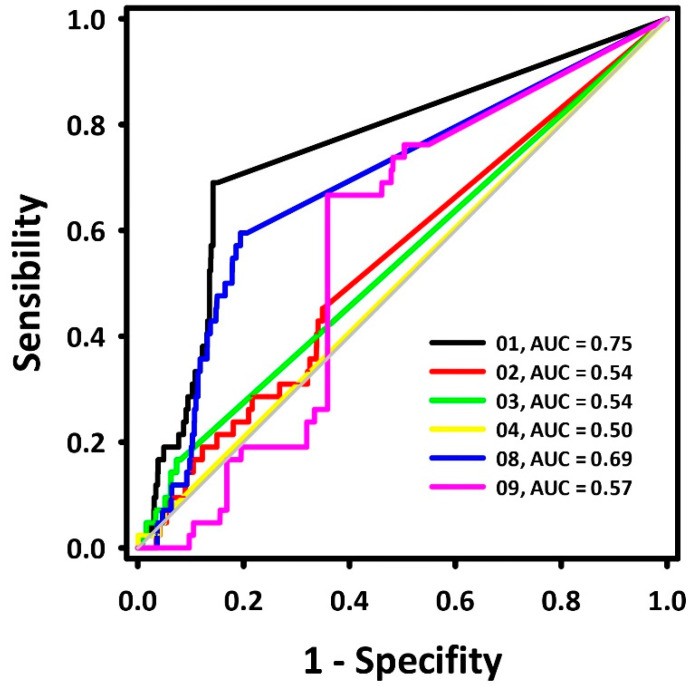
ROC curves for the BACE-1 inhibitor pharmacophore models.

A dataset with 42 BACE-1 inhibitors (IC_50_ ≤ 1000 nM) and 2100 decoys was aligned to each pharmacophore model. Their superposition value (QFIT value; 0–100) was employed to calculate the ROC curves, and their respective areas (area under the curve—AUC-ROC) were calculated [13]. The results are presented in Figure 1. 

The ROC curve displays the recognition of assets (represented by the Y coordinate) and false positives (represented by the X coordinate). An ideal curve first runs vertically along the Y axis, recognizing all assets, and then horizontally along the X axis continuously, which represents the recognition of all assets from the aligned molecule bank without any false positive recognition with its area under the curve (AUC) equal to 1.0 [13]. The diagonal line represents the ROC curve of a randomized trial, where pharmacophoric models with AUC < 0.50 are associated with models that perform worse than a random selection. In contrast, models with AUC > 0.70 are moderately predictive [14]. Previous research [15] describes hydrogen donor centers as essential requirements for the inhibition of BACE-1, while hydrogen acceptor centers define the potency of those inhibitors.

**Figure 2:**
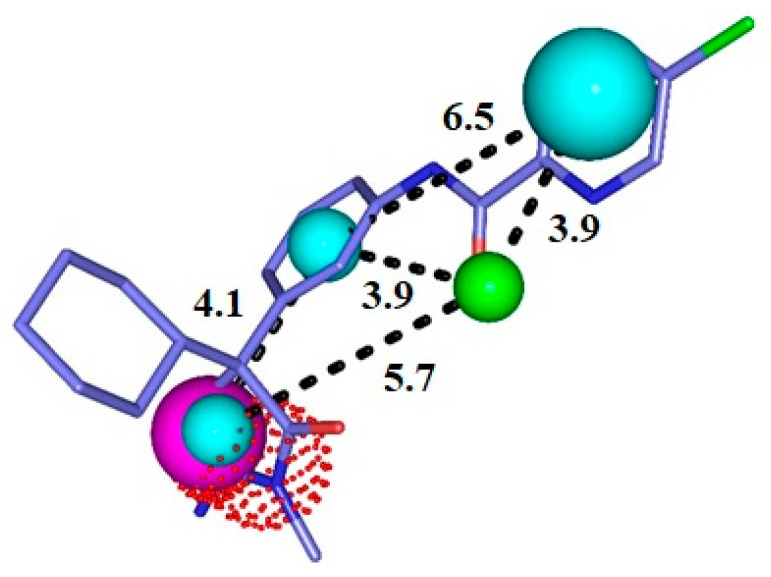
Representation of the pharmacophore model for BACE-1 inhibitors superimposed with a potent inhibitor. Cyan = hydrophobic centers (HY), red = positive centers, green = hydrogen acceptors (HBA), and magenta = hydrogen donors (HBD). The size of the spheres varies according to the tolerance radii calculated by GALAHAD™ (Seattle, WA, USA).

Based on the AUC values, pharmacophore model 1 (Figure 2) was chosen for further pharmacophore-based virtual screening. Its AUC-ROC was higher than 0.7, which characterized it to be the model with the highest potential capacity to correctly recognize active compounds. 

### 2.2. Pharmacophore-Based Virtual Screening

**Scheme 1:**
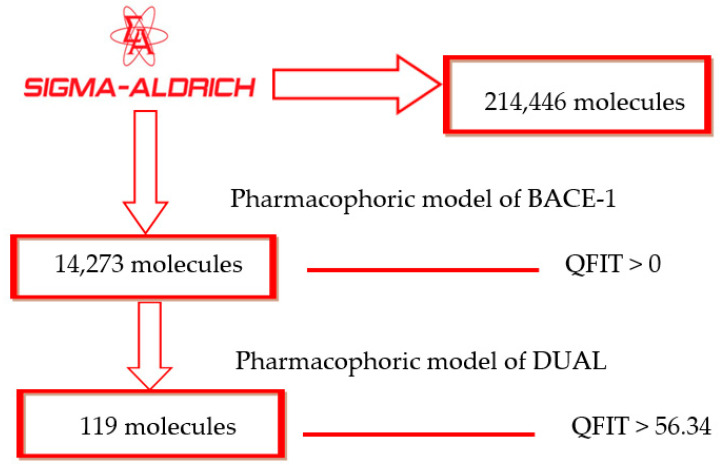
Virtual screening by the pharmacophore model.

After identifying a useful pharmacophore model, successive filtering of 214,446 molecules from a Sigma Aldrich^®^ dataset was performed. In total, 14,273 molecules showed QFIT > 0 when aligned to the BACE-1 inhibitor pharmacophore model 1. Subsequently, those molecules were filtered through the dual pharmacophore model previously built [7], with 119 molecules showing QFIT > 56.34 (Scheme 1), suggesting that they have stereo-electronic features important to biological activity. After virtual screening through pharmacophore models and based on alignment values, the selected structures were submitted to molecular docking studies with AChE, BChE, and BACE-1.

Although the pharmacophore model is helpful in searching for and selecting molecules that meet essential molecular requirements for biological activity [16], it has some limitations, such as the lack of information on how the molecules bind to the target site, as well as the limit imposed by the volume of the site. Those gaps in knowledge can be filled when the three-dimensional structure of the macromolecular target is available. The optimized application of molecular docking can also be used to assist in prioritizing bioactive molecules [17].

### 2.3. Molecular-Docking-Based Virtual Screening

Structures selected through pharmacophore model virtual screening (n = 119) were subjected to molecular docking against AChE, BChE, and BACE-1 using two different systems. AutoDock Vina, selected for molecular docking to AChE and BChE [7], scores structures by mapping intermolecular forces in kcal/mol, with lower energy indicating better docking. GOLD (ASP score function) was selected for molecular docking to BACE-1 [18]. It assigns a dimensionless number to each pose generated, and unlike AutoDock Vina, higher numbers indicate better docking.

The docking of the cholinesterases presented an average of the AChE energy values of −9.1, with 55 molecules showing lower affinity energy values. The BChE showed mean values of −10.02, with 67 molecules showing lower affinity energy values. The docking of BACE-1 showed mean energy values of 36.06, with 66 molecules presenting higher affinity energy values.

Then, the 22 molecules presenting the best values overall were selected and analyzed in order to exclude enantiomers. They were further evaluated based on their molecular coupling (or bonding) and on the presence of chiral centers. Three compounds, presenting the best values overall, were selected for further evaluation (Table 2).

In order to confirm our results, we re-ran the molecular docking of those three compounds against the three targets using the same programs. In other words, we ran ZINC45068352, ZINC03873986, and ZINC71787288 against AChE, BChE, and BACE-1 using AutoDock Vina 1.1.2 (despite it not being validated for BACE-1) and again, using GOLD 5.8.1, despite it not being validated for cholinesterases. The results, presented in the Supplementary Materials (Tables S1 and S2) confirm all three compounds present good scores in both programs, justifying their prioritization. 

### 2.4. Application of Physicochemical Filters

Good scores in molecular docking do not guarantee the selected molecules will have the physicochemical requirements to reach the target site. Therefore, the best candidates were further screened for their physicochemical properties according to Lipinski’s Rules and Veber’s parameters [19,20], which are capable of virtually predicting oral bioavailability quickly and at a low computational cost. The results are presented in Table 3.

Table 3 shows that the selected structures (ZINC45068352, ZINC03873986, ZINC71787288) satisfy all parameters for oral bioavailability according to Lipinski’s and Veber’s criteria, with only one suffering a penalty (ZINC71787288, with a cLogp value > 5). This penalty, however, does not justify eliminating the structure from consideration, as about 6% of the drugs orally bioavailable, currently in use, do not fully obey the accepted parameters for bioavailability [21]. 

### 2.5. Analysis of Intermolecular Interactions

Despite the importance of a selected structure overlapping the pharmacophore model, obtaining a good score in molecular docking, and having the physicochemical requisites for oral bioavailability, these metrics do not identify the bonds between potential inhibitors and their targets. Three-dimensional complexes were generated based on the known crystallographic structures of AChE, BChE, and BACE-1 to study the molecular bonds and mode of interaction of the three best-ranked compounds with the enzymatic active sites. 

#### 2.5.1. AChE Complexes

**Figure 3:**
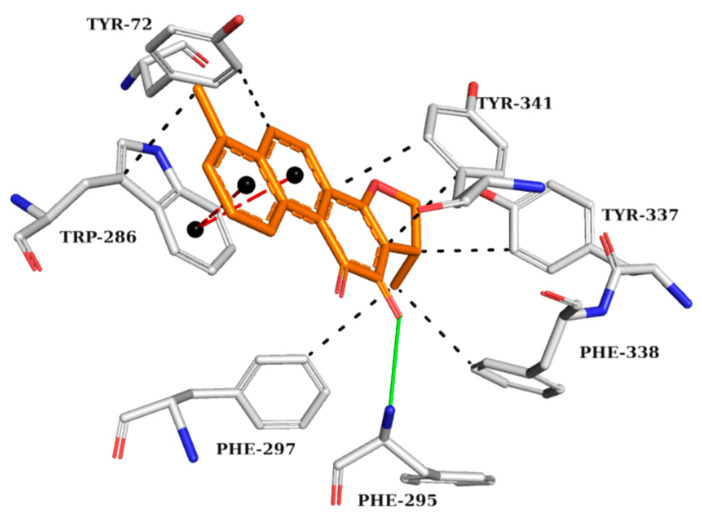
Interaction map of AChE crystallographic ligand (1YL—Dihydrotanshinone I) in the active site from PDB (4M0E), generated by the Protein–Ligand Interaction Profiler online server. Hydrogen bond as donor in solid green line, π stacking interactions in dashed red lines, and hydrophobic interactions in dashed black lines. (Orange: carbon of the ligand; blue: nitrogen; red: oxygen; gray: carbon of the amino acids; red: oxygen).

The analysis of the interactions performed by the AChE crystallographic inhibitor (Figure 3) can be useful in understanding the interactions important for biological activity and mapping those interactions for the subsequent analysis of the drug candidates screened by previous computational methods.

The AChE crystallographic ligand forms a hydrogen bond with PHE295, a π-stacking interaction with TRP286, and hydrophobic interactions with TYR72, TRP286, PHE297, TYR337, PHE338, and TYR341 (Figure 3).

**Figure 4:**
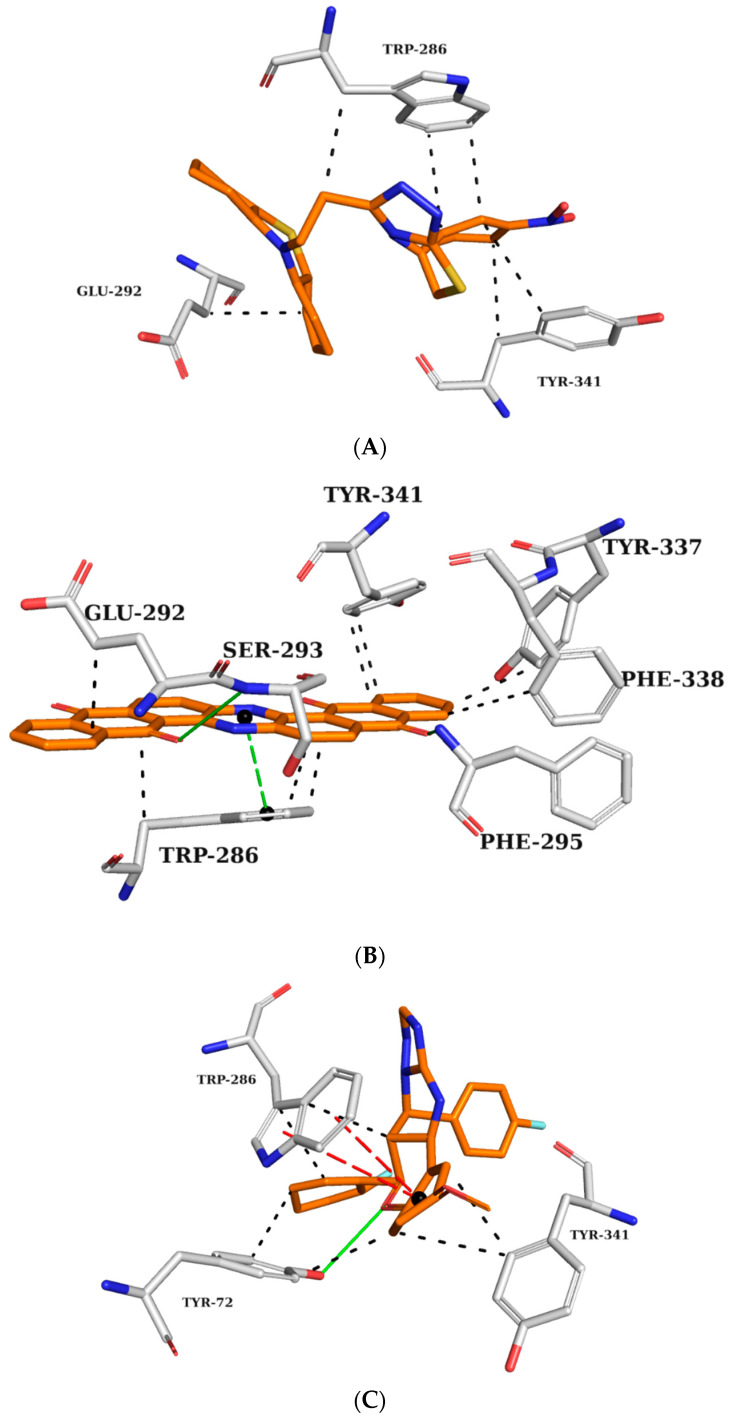
Interaction map of ZINC45068352 (**A**), ZINC03873986 (**B**), and ZINC71787288 (**C**) in the AChE active site generated by the Protein–Ligand Interaction Profiler online server. (The information is contained in Figure 3).

Based on this figure, interaction maps were generated for ZINC45068352 (Figure 4A), ZINC03873986 (Figure 4B), and ZINC71787288 (Figure 4C) in the AChE active site to observe whether the prioritized molecules maintained the same intermolecular interaction profile as the crystallographic ligand.

ZINC45068352 (Figure 4A) showed hydrophobic interactions at the AChE active site with TRP286 and TYR341, similar to the crystallographic ligand and additionally with the residue GLU292. ZINC03873986 (Figure 4B) repeated important interactions established by the crystallographic ligand, such as the hydrogen donor to PHE295, π-stacking interaction with TRP286, and hydrophobic interactions with TRP286, TYR337, PHE338, and TYR341. Furthermore, this molecule established π-stacking interactions with TYR341 and hydrophobic interactions with LEU76. ZINC71787288 (Figure 4C) made hydrophobic interactions with residues TYR72, TRP286, and TYR341, hydrogen bonding with TYR72, and π-stacking interactions with TRP286. Notably, the binding with TRP286 is related to potent compounds at the nanomolar scale, which also interact with the PHE 338 residue and participate in the binding of the substrate to the enzyme, ensuring catalytic efficiency [22,23,24].

#### 2.5.2. BChE Complexes

**Figure 5:**
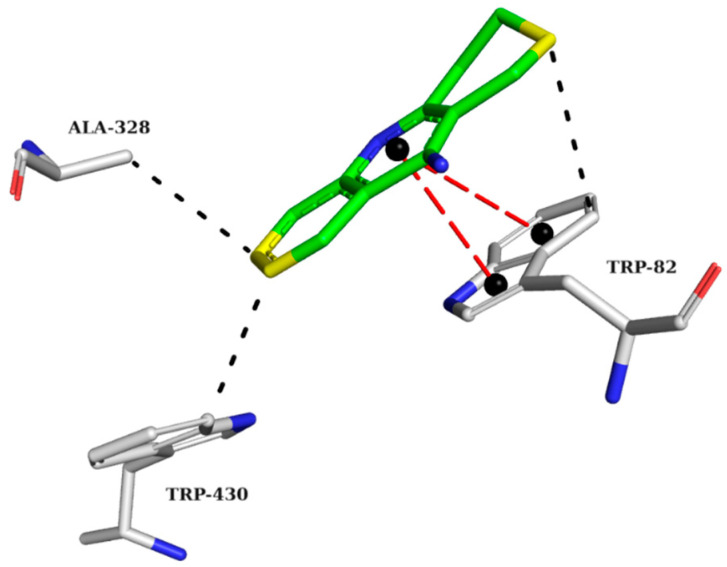
Interaction map of the BChE crystallographic ligand (THA) in the active site from PDB (4BDS). (The information is contained in Figure 3). (Green: carbon of the ligand; blue: nitrogen; red: oxygen; gray: carbon of the amino acids; red: oxygen).

The crystallographic structure of BChE with its inhibitor was analyzed to highlight the requirements for the proper interactions of the three best-ranked compounds. The interaction map of the BChE crystallographic ligand shows π-stacking interactions with TRP82 and hydrophobic interactions with TRP82, ALA328, and TRP430 (Figure 5).

**Figure 6:**
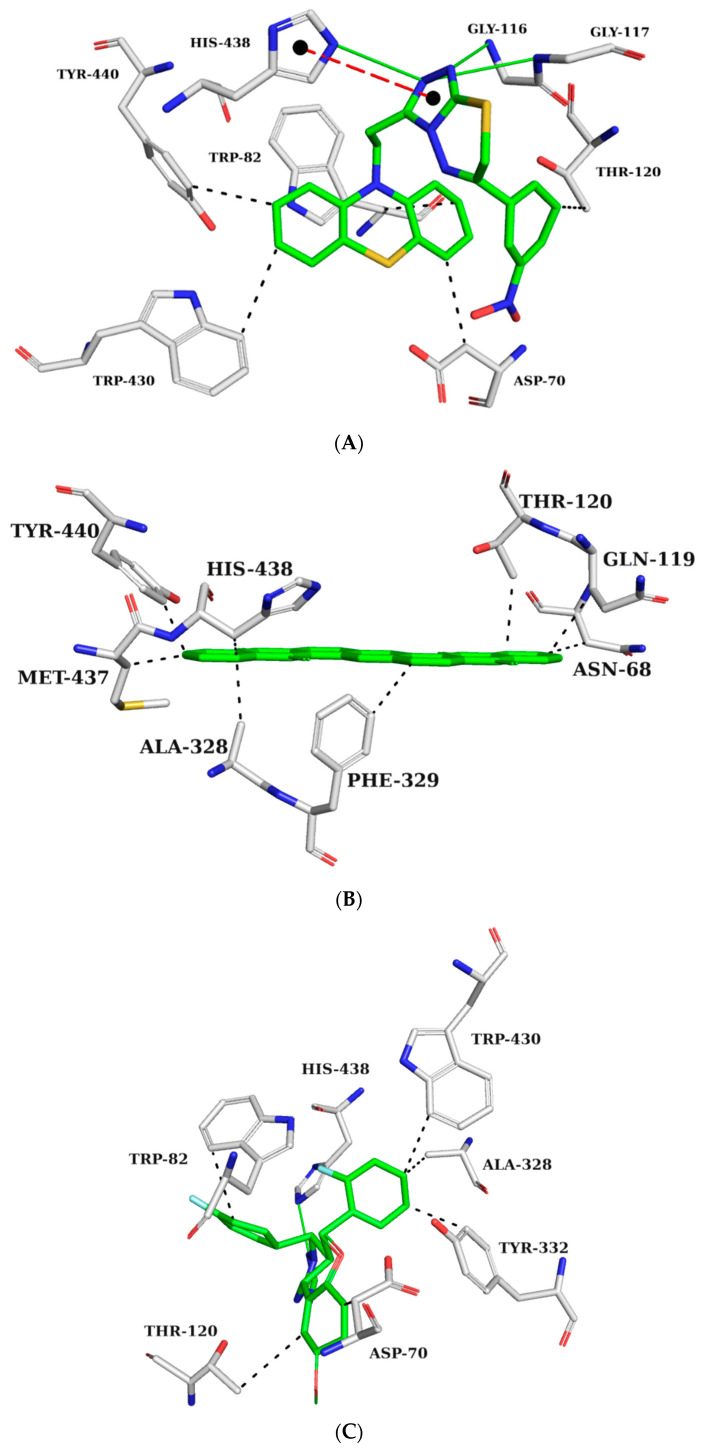
Interaction map of ZINC45068352 (**A**), ZINC03873986 (**B**), and ZINC71787288 (**C**) with the BChE active site generated by the Protein–Ligand Interaction Profiler online server. (The information is contained in Figure 5).

Interaction maps were subsequently generated for ZINC45068352 (Figure 6A), ZINC03873986 (Figure 6B), and ZINC71787288 (Figure 6C) to compare the interactions observed with the crystallographic ligand with the expected interactions with the selected molecules.

ZINC45068352 forms hydrogen bonds with GLY116, GLY117, HIS438, π-stacking interaction with HIS438, and hydrophobic interactions with residues ASP70, TRP82, THR120, TRP430, and TYR440 (Figure 6A). ZINC03873986 forms hydrophobic interactions with residues ASN68, ASP70, TRP82, and THR120 (Figure 6B). ZINC71787288 forms hydrogen bonds with HIS438 and hydrophobic interactions with ASP70, TRP82, THR120, ALA328, TYR332, and TRP430 (Figure 6C). The interactions observed with the TRP82 residues in the anionic site prevent the substrate from reaching the catalytic site. In the interaction maps, it is also possible to observe binding with residue ASP70, which is part of the peripheral site. This interaction is also important for inhibitory activity against BChE since it also prevents the entry of the substrate [25]. Interactions with residues THR120, GLY116, and GLY117 were observed in potent BChE inhibitors [26,27]. 

#### 2.5.3. BACE-1 Complexes

**Figure 7:**
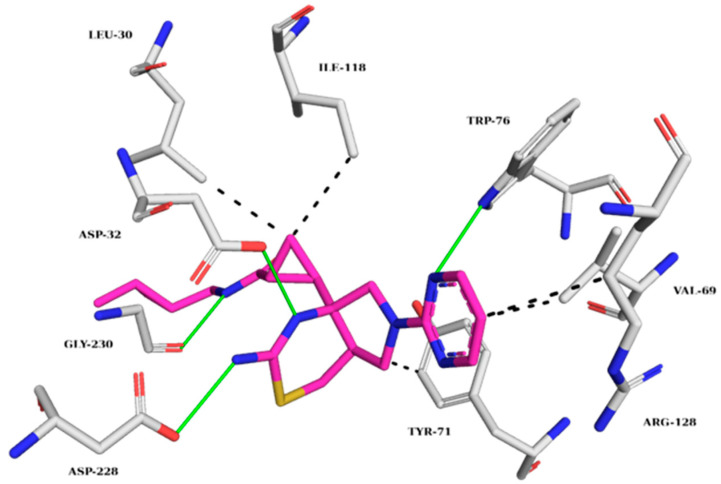
Interaction map of the BACE-1 crystallographic ligand (QKA) in the active site from PDB (6UWP). (The information is contained in Figure 3). (Margin: carbon of the ligand; blue: nitrogen; red: oxygen; gray: carbon of the amino acids; red: oxygen).

**Figure 8:**
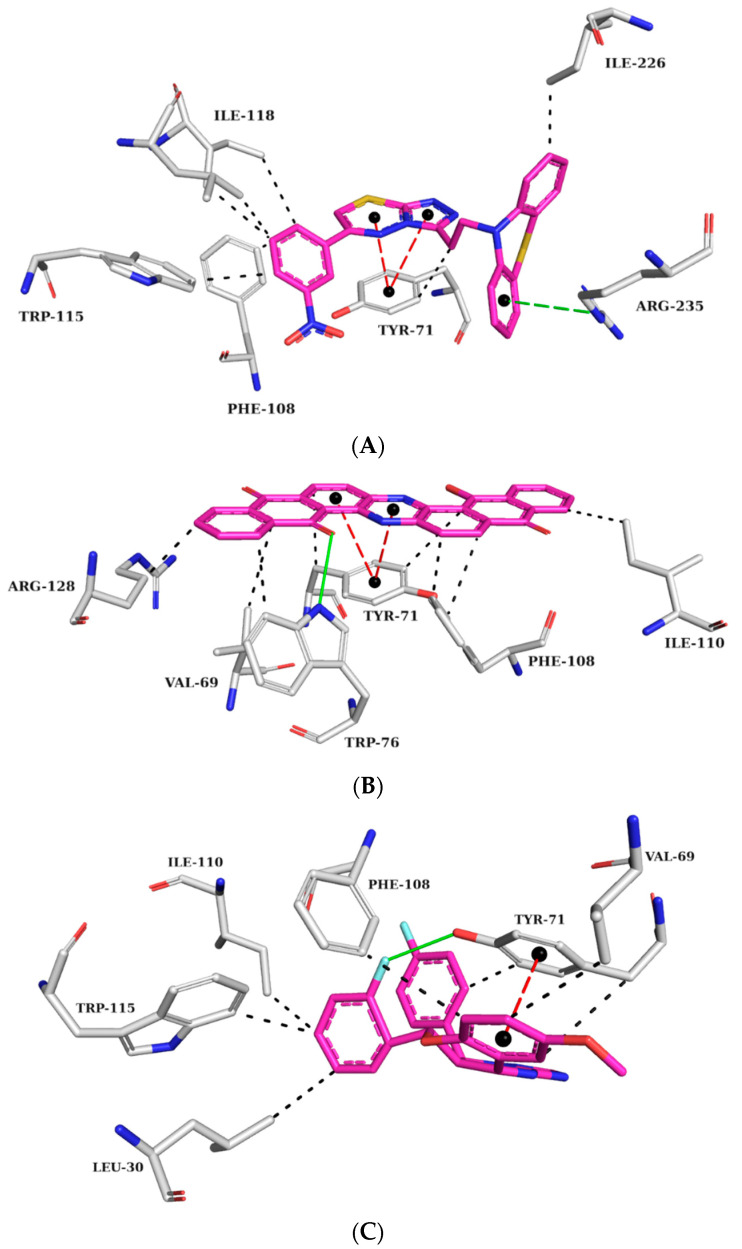
Interaction map of ZINC45068352 (**A**), ZINC03873986 (**B**), and ZINC71787288 (**C**) in the BACE-1 active site generated by the Protein–Ligand Interaction Profiler online server. (The information is contained in Figure 7).

The BACE-1 crystallographic ligand (Figure 7) forms hydrogen bonds with TRP76, ASP32, ASP228, and GLY230 and hydrophobic interactions with LEU30, VAL69, TYR71, ILE118, and ARG128 [18]. Figure 8 shows the analysis of the complexes generated between BACE-1 and ZINC45068352, ZINC03873986, and ZINC71787288.

At the BACE-1 active site, ZINC45068352 held π-stacking interactions with TYR71, hydrophobic interactions with residues TYR71, PHE108, TRP115, ILE118, and ILE226, and π-cation interactions with ARG35 (Figure 8A). As for ZINC03873986, it formed a hydrogen bond with TRP76, π-stacking interactions with TYR71, hydrophobic interactions with residues VAL69, TYR71, TRP76, PHE108, ILE118, and ARG128, and π-cation interactions with ARG35 (Figure 8B). ZINC71787288, displayed π-stacking interactions with TYR71, hydrogen bonding with TYR71, and hydrophobic interactions with residues LEU30, VAL69, TYR71, PHE108, ILE110, and TRP115 (Figure 8C). The published data showed that the observed interactions with the amino acid residue TYR71 are essential for inhibiting activity, as they promote conformational changes and prevent the substrate from reaching the catalytic site [28,29]. Interactions with amino acid residues VAL69, ILE118, and TRP115 are cited as important for BACE-1 inhibition, which has been observed in the molecular dynamics simulations already described [30]. 

### 2.6. Analysis of AMES Test (Cytotoxicity) and Other Parameters of Toxicity

After the molecular docking stage and the evaluation of their interactions with the three target enzymatic sites, the three compounds ZINC45068352, ZINC03873986, and ZINC71787288 were subjected to an in silico AMES test, using the online server pkCSM [29]. The AMES test is an essay originally performed on *Salmonella typhimurium* and *Escherichia coli*. It is based on the knowledge that if a substance is mutagenic for these bacteria, it also presents a risk of developing cancer in humans [31]. In silico mutagenicity screening tools were later developed and optimized to screen drug candidates, yielding comparable results to the original AMES test [32,33,34]. 

ZINC03873986 and ZINC71787288 were negative for the AMES test, but ZINC45068352 was positive and discarded from future steps. Additional simulations were performed for the structures ZINC03873986 and ZINC71787288 to characterize other aspects of their toxicity profile. According to our analysis, presented in the Supplementary Materials (Table S3), ZINC71787288 is hepatotoxic, which leaves ZINC0387398 as our lead compound.

## 3. Materials and Methods

### 3.1. Dataset

**Figure 9:**
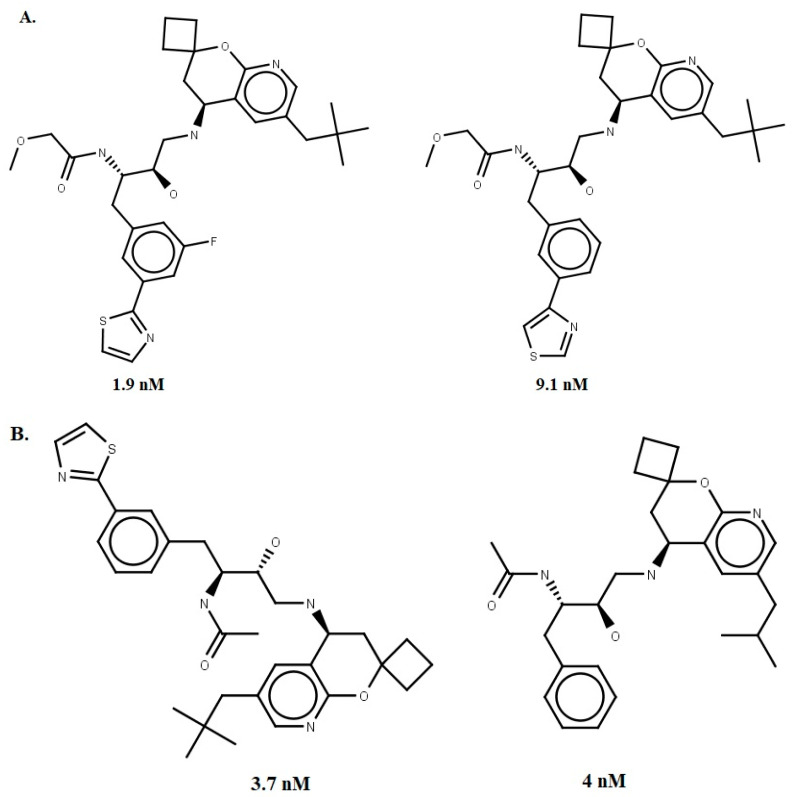
The four chemical structures and biological activities of inhibitors against BACE-1 that were used in generating (**A**) and evaluating (**B**) the pharmacophore models.

A dataset of 56 compounds (Supplementary Materials Tables S4 and S5) with IC_50_ ≤ 1000 nM for human BACE-1 was obtained from the literature [35]. The 2D structures and most reliable tautomers (pH = 4.5) were drawn using Marvin^®^ Sketch 15.4.20 [36]. Subsequently, the structures were converted to 3D format using the CONCORD module, implemented in the SYBYL^®^-X 2.0 package [37]. Partial atomic charges were calculated using the Gasteiger–Hückel method, as available on the SYBYL platform. Energy minimization was performed through Conjugate Gradient (CG) with a convergence criterion of 0.001 kcal/mol and Tripos force field (dielectric constant ε = 80.0 and a maximum number of iterations = 50.000) [12]. Four compounds with the best IC_50_ values were selected, two for the construction and two for the validation stage of the pharmacophore model (Figure 9).

### 3.2. Pharmacophore Model Generation

The GALAHAD (Genetic Algorithm Linear Algorithm for Hyper molecular Alignment of Data sets) implemented on the SYBYL platform was used to obtain the conformers. The flexibly superimposed training set compounds generated the pharmacophore features to create hyper molecular alignments. The Genetic Algorithm (GA) employed in this step starts with 80 conformations (population size) of each compound that evolves through a maximum of 830 generations. The other parameters (CROSSING = 1.0 and MUTATION = 1.0) were maintained at their default values, as implemented in the GALAHAD module from SYBYL-X^®^ 2.0 [37].

### 3.3. Pharmacophore Model Evaluation 

The statistical parameters of GALAHAD (ENERGY < 100 Kcal/mol and PARETO ≠ 00) were used to select the pharmacophore models. The discriminatory power to recognize active compounds and decoys evaluated the remaining models. Thus, the DUD-E server [38] was used to build decoys, and the SigmaPlot^®^ program v. 12.0 [39] was used to calculate the area under the curve of each receiver operating characteristic (AUC-ROC curve). The model that attained an AUC-ROC > 0.7 was chosen as the best BACE-1 inhibitor pharmacophore model.

### 3.4. Pharmacophore-Based Virtual Screening

The best BACE-1 pharmacophore model was used to filter the database Sigma-Aldrich^®^ (n = 214,446) (http://zinc15.docking.org/catalogs/sialbb accessed on 5 September 2020) available on the ZINC15 platform [40] by using the UNITY module of SYBYL-X 2.0. This step was implemented through the option “3D flexible alignment”, available in the UNITY 3D module. The quality of the alignment of the molecules was expressed by the value of QFIT, which can vary from 0 to 100.

The superimposed compounds in the BACE-1 model (QFIT > 0) were then flexibly aligned with a dual AChE and BChE inhibitors pharmacophore model [7], available in the GALAHAD™ module. To prioritize the best-superimposed compounds in this model, the mathematical equation of average plus the standard deviation of QFIT values (Equation (1)) was employed as a cutoff. Compounds showing QFIT > $\underline{x}$ + σ were then selected for molecular docking with AChE, BChE, and BACE-1. Equation (1) was used to select the compounds best fitting the dual pharmacophore model:

(1) $$X\;\geq \;\underline{x}+\sigma ,$$

where X= QFIT value, $\underline{x}=$ average, and σ = standard deviation.

### 3.5. Molecular Docking

The crystallographic structures of AChE (PDB ID: 4M0E) [22], BChE (PDB ID: 4BDS) [41], and BACE-1 (PDB ID: 6UWP) [42] were prepared with Biopolymer implemented on SYBYL-X 2.0 [37], where ions and water molecules were removed. Hydrogen atoms were inserted to optimize the hydrogen bonds. For the AChE and BChE target structures, the protonation state of the receptors was adjusted to pH 7.4 through the PropKa [43] server, and the conformational search and scoring were performed by AutoDock Vina 1.1.2 [44], according to previously validated parameters [7]. As for the BACE-1 structure, the receptor had its protonation state evaluated by the H++ 1.0 server [http://newbiophysics.cs.vt.edu/H++/ accessed on 24 September 2020] program, and pKa was corrected at pH 4.5 [45]. Validation methods were used, and the program selected for molecular docking with BACE-1 was GOLD 5.8.1 [46]; the score was provided by the Astex Scoring Potential (ASP, knowledge-based function derived from a database of protein–ligand complexes) function with the parameters previously validated [18]. 

### 3.6. Physicochemical Filters 

The designed molecules were characterized using the pkCSM server [34] for the physicochemical descriptors for Lipinski’s [19] and Veber’s [20] descriptors. For reference:

- Lipinski’s: Molecular Weight (MW) ≤ 500 Da; Hydrogen Bond Donors (HBD) ≤ 5; Hydrogen Bond Acceptors (HBA) ≤ 10; and cLog*p* ≤ 5.
- Veber’s: Polar Surface Area (PSA) ≤ 140 ${Å}^{2}$; Rotatable Bonds (RB) ≤ 10; Sum of HBD and HBA ≤ 12.

### 3.7. Evaluation of Intermolecular Interactions

Molecules having one or fewer penalties were selected for evaluation of the intermolecular interaction through the Protein–Ligand Interaction Profiler (PLIP) server and PyMOL v. 2.4.0 [47,48].

### 3.8. AMES Test (Cytotoxicity) and Other Parameters of Toxicity

After the molecular docking stage and evaluation of active-site interactions, the three higher-scoring compounds were subjected to the AMES test [31] to predict their potential cytotoxicity using the pkCSM server [34]. The results led to ZINC45068352 being discarded. The same pkCSM server was used to analyze other parameters of toxicity, such as oral rat acute toxicity (LD_50_), hepatotoxicity, and skin sensitization, among others. The results, presented in Table S3 of the Supplementary Materials, indicated that ZINC71787288 was hepatotoxic, leading to it also being discarded.

## 4. Conclusions

The use of multitarget drugs is relatively recent in the history of therapy, with those few showing distinct advantages over a combination of separate drugs. We expect a multitarget treatment against AD would need lower doses, present fewer drug–drug interactions, and encourage higher patient compliance.

The virtual screening strategy associated with the individual pharmacophore models for AChE, BChE, and BACE1 allowed the generation and evaluation of models evaluated for recovery rate of true inhibitors versus false positives, resulting in the selection of a pharmacophore model with discriminatory power (AUC > 0.7). This approach, aligned with a dual pharmacophore model and docking, allowed the identification of possible hybrid triple-inhibitors against AChE, BChE, and BACE1. Computational techniques employed in a hierarchical process enabled the selection of molecules with proper stereo-electronic requirements for triple-target inhibition. 

ZINC03873986 was selected as a good fit for the pharmacophore models with low cytotoxic potential, which makes it a potential multitarget hybrid compound for the treatment of Alzheimer’s disease. Our next steps aim to validate these computational results through enzymatic testing on the targets and synthesis of derivatives of those lead compounds for SAR studies.

## Figures and Tables

**Table 1:** Internal statistical parameters of each pharmacophore model for the BACE-1 inhibitors provided by GALAHAD.

Model	Energy (Kcal/mol)	Sterics	H_Bond	Mol_qry	Pareto
01	21.74	756.00	155.60	9.09	00
02	42.36	746.60	151.50	15.59	00
03	20.65	687.30	157.00	14.42	00
04	50.89	713.40	161.20	8.47	00
05	1043.58	746.90	153.00	14.40	00
06	783.55	738.00	161.30	6.34	00
07	151,768.74	779.70	152.30	15.64	00
08	64.82	750.30	161.20	2.92	00
09	25.54	693.90	155.80	9.04	00
10	254.77	723.20	153.60	10.54	00

**Table 2:** Scores of the three best compounds selected through molecular docking.

Molecule	AChE *	BChE *	BACE-1 **
ZINC45068352	−9.6	−10.4	43.32
ZINC03873986	−10.6	−11.2	41.54
ZINC71787288	−9.6	−10.6	41.91

* AutoDock Vina 1.1.2—kcal/mol—lower energy indicates better docking. ** GOLD—dimensionless—Higher numbers indicate better docking.

**Table 3:** Physicochemical analysis of the best candidates for triple inhibition according to Lipinski’s Rule and Veber’s parameters.

Molecule	MW (g/mol)	HBD	HBA	cLogP	$\mathrm{PSA}\;({Å}^{2})$	RB	HBD + HBA
ZINC45068352	486.58	0	7	4.77	89.45	5	7
ZINC03873986	442.43	2	6	4.15	100.27	0	8
ZINC71787288	444.44	0	7	5.38	61.53	3	7

MW = molecular weight; HBD = hydrogen bond donor; HBA = hydrogen bond acceptor; cLogP = calculated octanol–water partition coefficient; PSA = Polar surface area; RB = Rotatable bonds.

## Data Availability

Data is contained within the article and supplementary material.

## Supplementary Materials

The following supporting information can be downloaded at: https://www.mdpi.com/article/10.3390/ph16121645/s1, Table S1. Scores of the three best compounds selected through molecular docking by AutoDock Vina 1.1.2. Table S2. Scores of the three best compounds selected through molecular docking by GOLD. Table S3. Toxicological analysis of the remaining molecules. Table S4. Chemical structure and biological activity of inhibitors against BACE-1 used in generating pharmacophore models. Table S5. Chemical structure and biological activity of inhibitors against BACE-1 that were used in evaluating pharmacophore models.
