# Supplementary material for: Identification of Potential Multitarget Compounds against Alzheimer’s Disease through Pharmacophore-Based Virtual Screening
Source: Pharmaceuticals (Basel). 2023 Nov 23;16(12):1645. doi: 10.3390/ph16121645 (PMC10748159; doi:10.3390/ph16121645)
Supplement: Supplementary file 1 [file pharmaceuticals-16-01645-s001.zip › pharmaceuticals-2580844-supplementary.pdf]

Article

Identification of Potential Multitarget Compounds against Alzheimer's Disease through Pharmacophore-Based Virtual Screening

Géssica Oliveira Mendes [1], Moysés Fagundes de Araújo Neto [1], Deyse Brito Barbosa [1], Mayra Ramos do Bomfim [1], Lorena Silva Matos Andrade [2], Paulo Batista de Carvalho [3], Tiago Alves de Oliveira [4,5], Daniel Luciano Falkoski [4], Eduardo Habib Bechelane Maia [5], Marcelo Siqueira Valle [6], Laila Cristina Moreira Damázio [7], Alisson Marques da Silva [5], Alex Gutterres Taranto [4] and Franco Henrique Andrade Leite [1,*]

[1] Laboratory of Molecular Modeling, Department of Health, State University of Feira de Santana, Feira de Santana 44036-900 BA, Brazil; gomendes05@gmail.com (G.O.M.); moysesfagundes@gmail.com (M.F.d.A.N.); deyse.brito@hotmail.com (D.B.B.); mayramosbonfim@hotmail.com (M.R.d.B.)

[2] Laboratory of Chemoinformatics and Biological Assessment, Department of Health, State University of Feira de Santana, Feira de Santana 44036-900, BA, Brazil; lorena.pharm@gmail.com

[3] Feik School of Pharmacy, University of the Incarnate Word, San Antonio, TX 78212, USA; pcarvalh@uiwtx.edu

[4] Department of Bioengineering, Federal University of São João del-Rei, São João del-Rei 36301-160, MG, Brazil; tiagofga@cefetmg.br (T.A.d.O.); dlfalkoski@yahoo.com.br (D.L.F.); proftaranto@hotmail.com (A.G.T.)

[5] Department of Informatics, Management and Design, Federal Center for Technological Education of Minas Gerais (CEFET-MG), Divinópolis 35503-822, MG, Brazil; habib@cefetmg.br (E.H.B.M.); alisson@cefetmg.br (A.M.d.S.)

[6] Department of Natural Sciences, Federal University of São João del-Rei, São João del-Rei 36301-160, MG, Brazil; marcelovalle@gmai.com (M.S.V.)

[7] Department of Medicine, Federal University of São João del-Rei, São João del-Rei 36301-160, MG, Brazil; lailacmdamazio@gmail.com (L.C.M.D.)

[*] Correspondence: fhenrique@uefs.br (F.H.A.L.)

Table S1. Scores of the three best compounds selected through molecular docking by AutoDock Vina 1.1.2.

Molecule	AChE *	BChE *	BACE-1 *
ZINC45068352	-9.6	-10.4	-9.9
ZINC03873986	-10.6	-11.2	-12.2
ZINC71787288	-9.6	-10.6	-9.6

*kcal/mol - lower energy indicates better docking

Table S2. Scores of the three best compounds selected through molecular docking by GOLD.

Molecule	AChE *	BChE *	BACE-1 *
ZINC45068352	58.82	63.25	43.32
ZINC03873986	68.49	37.38	41.54
ZINC71787288	57.65	56.64	41.91

*dimensionless - Higher numbers indicate better docking

Table S3. Toxicological analysis of the remaining molecules.

Molecule	Maximum Tolerated dose (human)	hERG I inhibitor	Oral Rat Acute Toxicity (LD50)	Oral Rat Chronic Toxicity (LOAEL)	Hepatotoxicity	Skin Sensitization	T.Pyriformis toxicity	Minnow toxicity
ZINC03873986	0.695	No	2.499	3.126	No	No	0.286	-0.457
ZINC71787288	0.639	No	2.709	2.23	Yes	No	0.321	-0.361

Table S4. Chemical structure and biological activity of inhibitors against BACE-1 used in generating pharmacophore models.

Nº	Chemical structure	IC_{50} (nM)
1	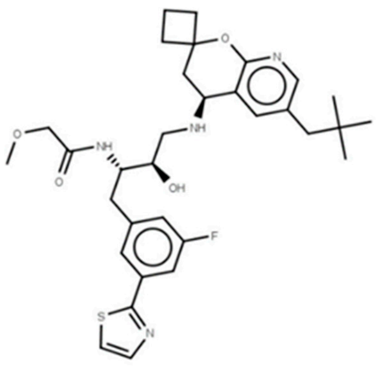	1.9
11	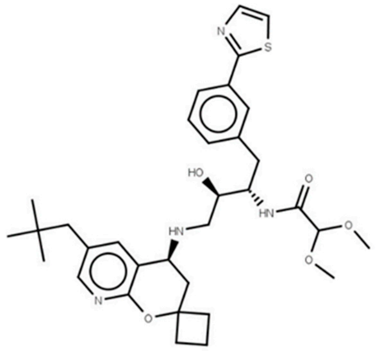	9.1
17	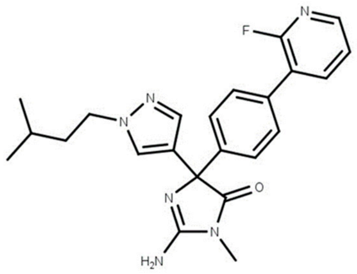	16
19	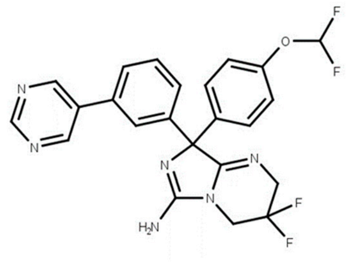	19.5

22	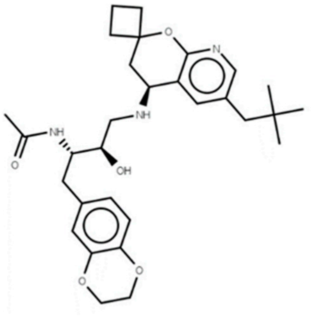	22.8
28	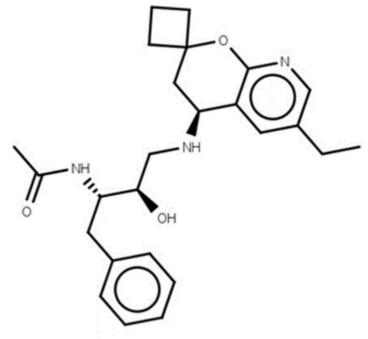	39
31	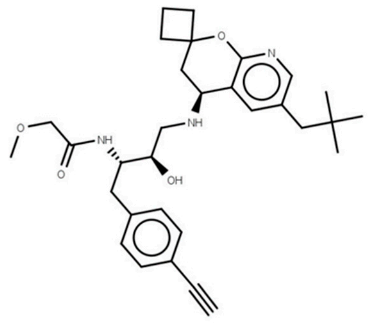	47
32	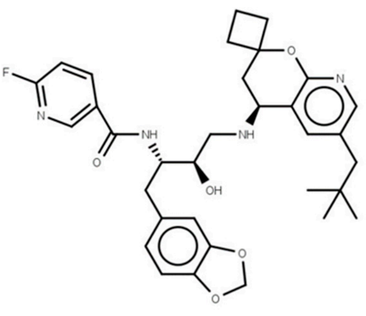	48
38	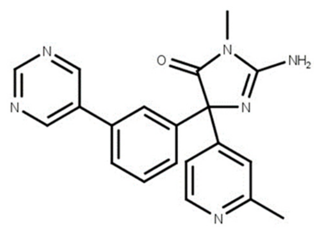	79.98

39	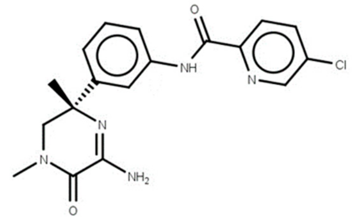	83
40	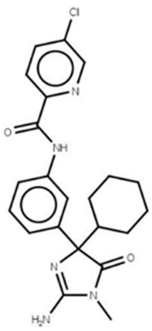	100
41	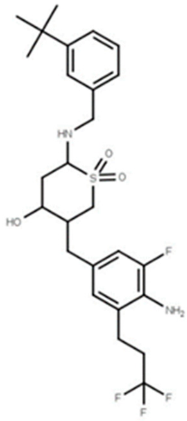	130
42	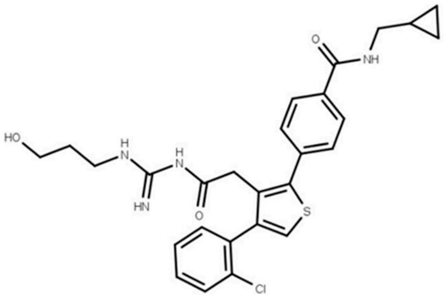	140
44	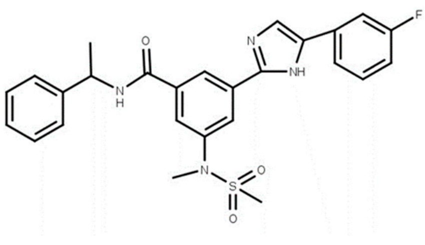	187

Table S5. Chemical structure and biological activity of inhibitors against BACE-1 that were used in evaluating pharmacophore models.

Nº	Chemical structure	IC_{50} (nM)
2	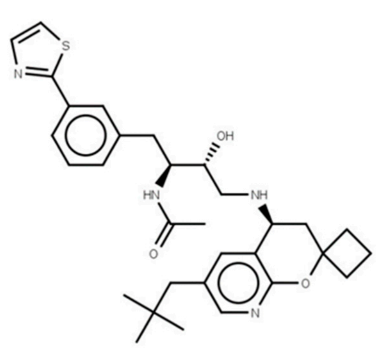	3.7
3	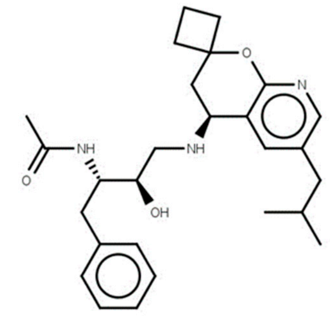	4
4	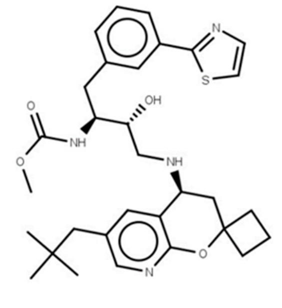	4.1
5	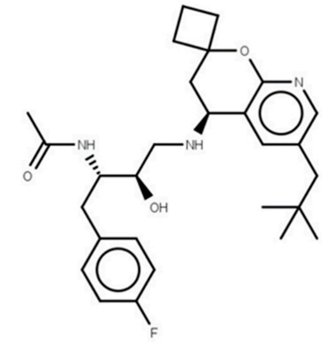	5

6	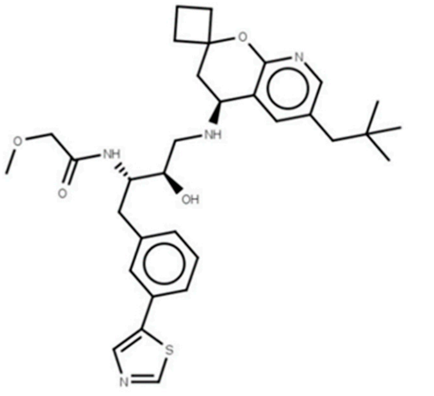	5.2
7	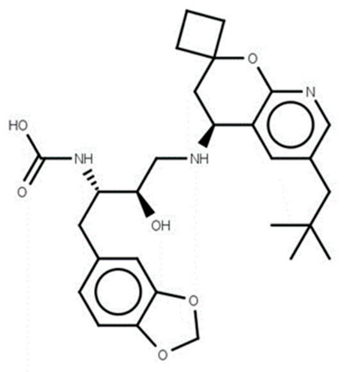	5.4
8	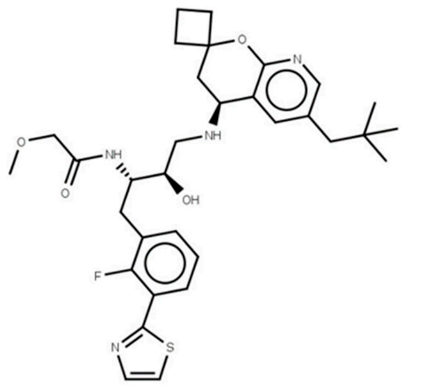	6.1
9	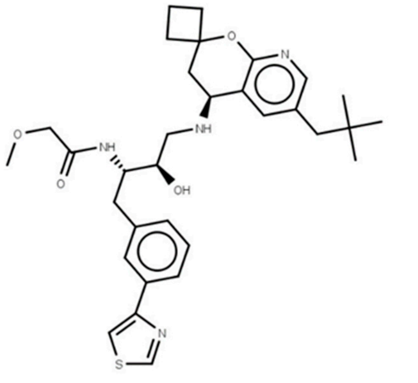	6.9

10	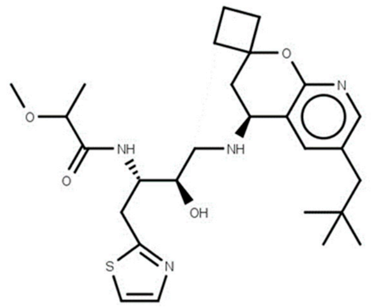	7.2
12	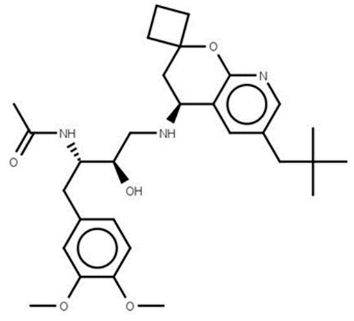	9.3
13	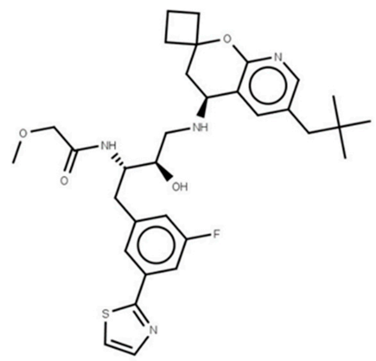	9.5
14	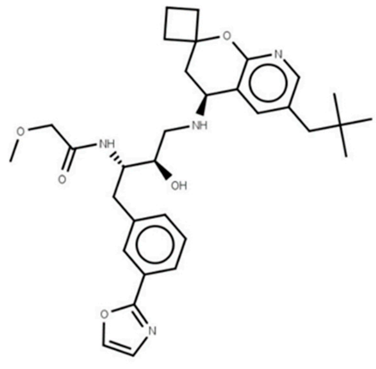	9.6
15	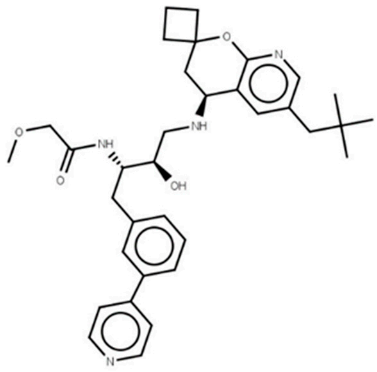	9.8

16	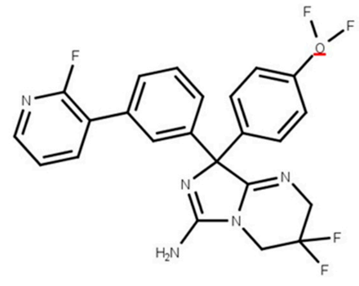	12.59
18	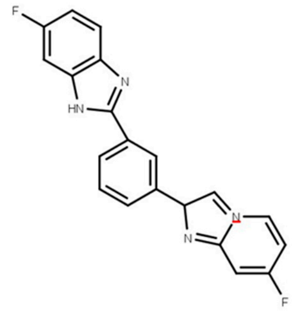	18
20	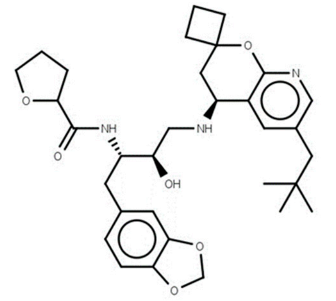	20.4
21	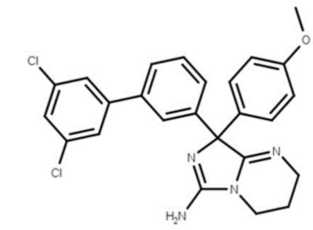	22.39
23	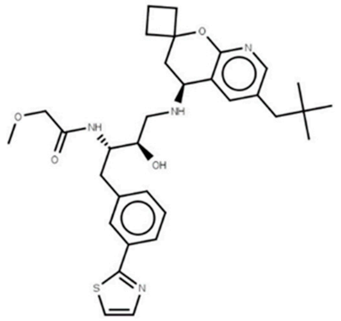	26

24	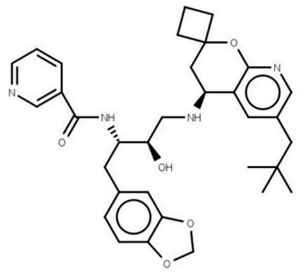	26.1
25	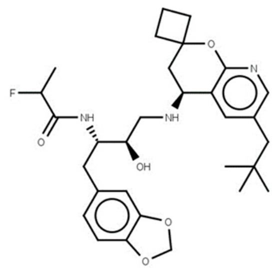	28.9
26	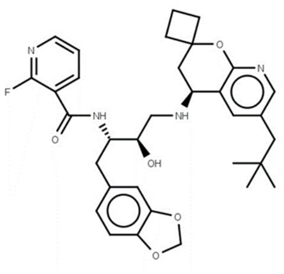	33.2
27	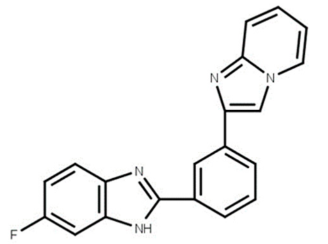	35
29	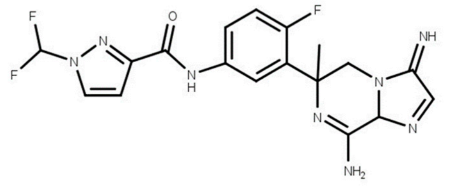	46.67
30	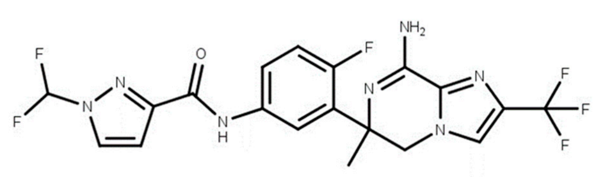	46.77
33	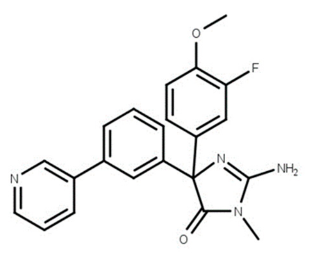	50

34	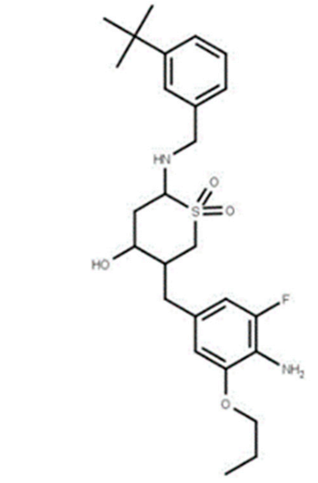	56
35	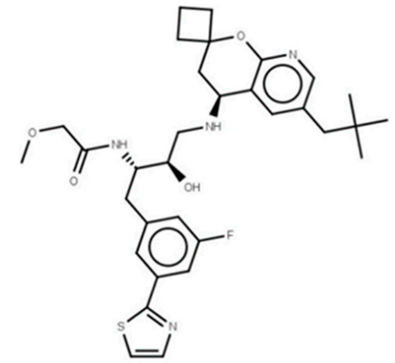	71
36	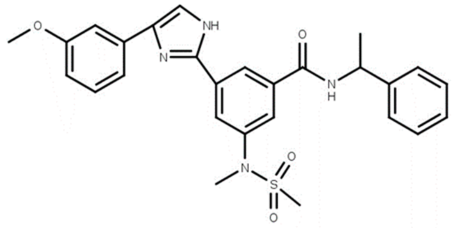	77
37	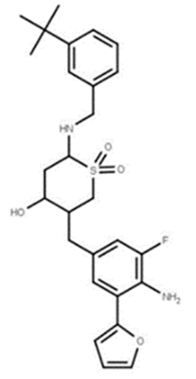	79

43	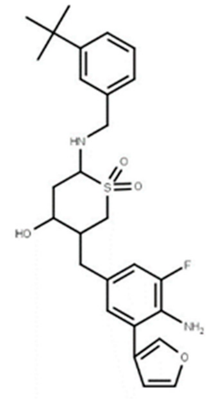	150
45	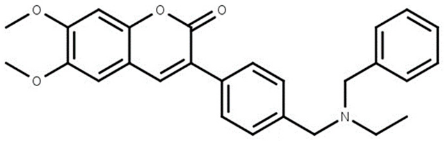	238
46	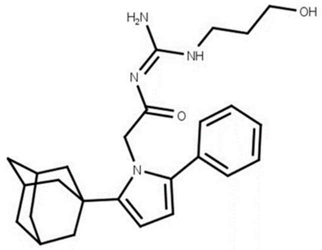	239
47	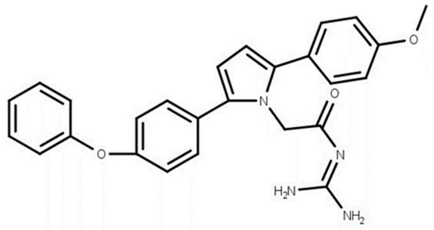	500
48	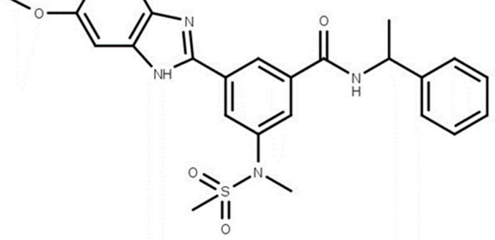	545
49	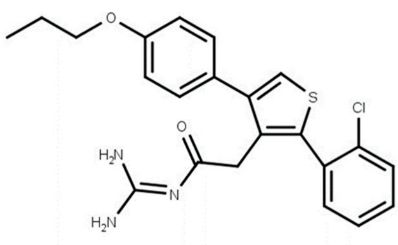	590

50	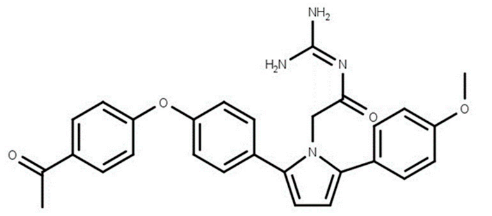	600
51	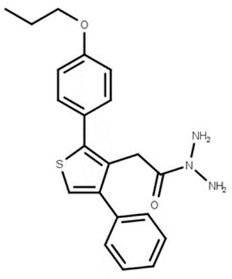	680
52	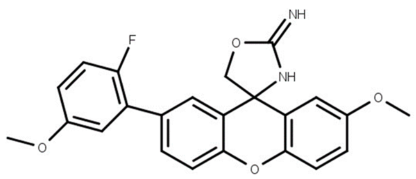	740
53	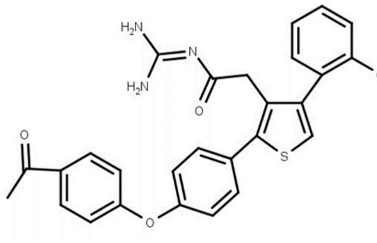	750
54	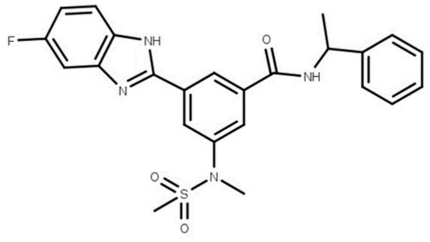	786
55	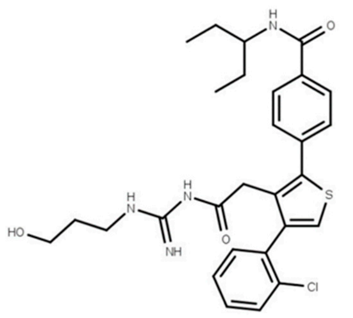	930

56	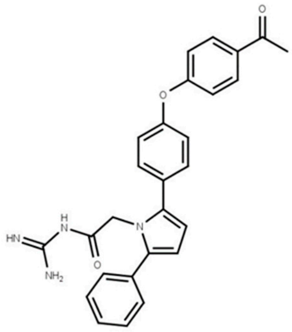	1000
